# Affective and motivational influences in person perception

**DOI:** 10.3389/fnhum.2013.00266

**Published:** 2013-06-11

**Authors:** Bojana Kuzmanovic, Anneli Jefferson, Gary Bente, Kai Vogeley

**Affiliations:** ^1^Institute of Neuroscience and Medicine - Ethics in the Neurosciences (INM-8), Research Center JuelichJuelich, Germany; ^2^Department of Psychiatry and Psychotherapy, University Hospital CologneCologne, Germany; ^3^Department of Psychology, University of CologneCologne, Germany; ^4^Institute of Neuroscience and Medicine - Cognitive Neurology (INM-3), Research Center JuelichJuelich, Germany

**Keywords:** person perception, impression formation, social inference, affective influence, reward

## Abstract

Interpersonal impression formation is highly consequential for social interactions in private and public domains. These perceptions of others rely on different sources of information and processing mechanisms, all of which have been investigated in independent research fields. In social psychology, inferences about states and traits of others as well as activations of semantic categories and corresponding stereotypes have attracted great interest. On the other hand, research on emotion and reward demonstrated affective and motivational influences of social cues on the observer, which in turn modulate attention, categorization, evaluation, and decision processes. While inferential and categorical social processes have been shown to recruit a network of cortical brain regions associated with mentalizing and evaluation, the affective influence of social cues has been linked to subcortical areas that play a central role in detection of salient sensory input and reward processing. In order to extend existing integrative approaches to person perception, both the inferential-categorical processing of information about others, and affective and motivational influences of this information on the beholder should be taken into account.

## Introduction

For humans, other people are one of the most important sources of joy and sorrow. Our perception of others has far-reaching consequences for immediate reactions as well as for the likelihood and nature of future interactions. Thus, in everyday life, impression formation is crucial not only for private relationships but also for decisions regarding economic or political affairs (Delgado et al., 2005; Uleman et al., 2008; Antonakis and Dalgas, 2009). Due to its relevance, “person perception” in its broadest sense, i.e., covering sensory, cognitive, and affective processing of information about others, has generated intense research interest in a variety of disciplines. This widespread interest is in part due to the fact that the perception of persons fundamentally differs from that of objects insofar as it involves recognition of some other as an epistemic and moral subject and the possibility of reciprocity (Sturma, 1997).

Empirical approaches toward person perception describe different sources of person-related information and different kinds of impact of this information on the decoder. Within the classic social psychological research on categorical representation of others and inferences about others' current mental states and enduring personality traits, the person-related cues are seen as providers of diagnostic knowledge (Kelley, 1967; Trope, 1986; Mitchell et al., 2006; Freeman et al., 2010b). But psychological and neuroscientific research also demonstrated that interpersonal social cues have an intrinsic affective and motivational value and are able to influence sensory, inference and decision processes, as well as response selection (Klin et al., 2003; Winkielman et al., 2005; Vuilleumier and Pourtois, 2007; Schilbach et al., 2010). The relative involvement of these well-documented inferential-categorical and affective-motivational processes in person perception, however, crucially depends on the specific information format, e.g., whether information is conveyed verbally or non-verbally (Freeman et al., 2010b; Zaki et al., 2010; Kuzmanovic et al., 2012).

Critically, all these different approaches to person perception do not refer to processes that run independently, rather, they reciprocally modulate each other and the final information integration (Vuilleumier and Pourtois, 2007; Pessoa, 2008; Freeman and Ambady, 2011; Freeman et al., 2012). Nevertheless, empirical and theoretical approaches mostly focus only on one selective aspect of person perception, and are only beginning to develop integrative and comprehensive models (Freeman and Ambady, 2011; Freeman et al., 2012). For instance, the dynamic interactive theory of person construal integrates insights from social psychology and functional neuroimaging research related to face-processing by emphasizing complex interactions of cognitive processes underlying initial activation of categories (and corresponding stereotypes) and further higher order social reasoning (Freeman and Ambady, 2011; Freeman et al., 2012). This theory provides an excellent framework based on a recurrent connectionist model (Freeman and Ambady, 2011) and does refer to “top-down influences that originate in the perceiver (e.g., existing knowledge structures and motivations) and (…) bottom-up influence of factors that originate in the target of perception (e.g., overlapping visual cues)” (Freeman et al., 2012, p. 3). However, while this framework considers bottom-up influences of sensory input on category and stereotype activation, it does not explicitly address affective and motivational properties of social stimuli resulting in enhanced and prioritized processing and reward-dependent learning effects. These effects have been extensively documented in the emotion and reward-related neuroimaging research (see section Affective Influences of Person-related Information on the Decoder), even in newborns without fully developed propositional knowledge (Farroni et al., 2002), and do not have to relate to categorical organization of social cognition that is described in the person construal theory (Vuilleumier and Pourtois, 2007). By delineating distinct approaches to person perception related to cognitive inferential-categorical processing (section Person-related Knowledge as a Basis for Social Reasoning) and affective-motivational influences (section Affective Influences of Person-related Information on the Decoder), respectively, and by specifying relative processing differences for verbal and non-verbal formats of information (section Processing Differences for Distinct Formats of Person-related Information), the present paper aims to extend existing integrative views on person perception by emphasizing the critical role of salience and reward-related effects within the dynamic processing of social others (section Integration of Distinct but Interactive Person Perception Aspects).

## Person-related knowledge as a basis for social reasoning

Traditionally, social psychology has been primarily interested in how we form high-level impressions of others and how knowledge about others is represented within a categorically organized semantic system (Freeman and Ambady, 2011). Originally, it was supposed that people are trying to causally explain the observed behavior of others. Attribution Theory defined conditions in which logical and objective reasoning leads to the assumption of internal, i.e., disposition-related, or external, i.e., situation-related, causes for actions, dependent upon the available information about the target person (Heider, 1958; Jones and Davis, 1965; Kelley, 1967). Novel approaches additionally integrate initial lower-level perceptual interpretation and categorization processes in order to account for top-down and bottom-up dynamic interactions within social reasoning (Freeman and Ambady, 2011). Such models comprehensively explain how categories and corresponding stereotypes along with individuating information are used to form impressions of others and to understand their personality characteristics and current mental states. Furthermore, the general ability to attribute mental states such as beliefs and intentions to oneself and others in order to understand and predict behavior has been investigated with reference to “theory of mind” (ToM) (Premack and Woodruff, 1978). A prominent way to assess this ability is to use “false belief tasks” with social scenarios or non-verbal cues where test persons have to differentiate between their own perceptions, attitudes, or beliefs from those of others (Wimmer and Perner, 1983). Neuroimaging studies were able to associate this inferential and category-based social processing with a network consisting of the medial prefrontal, the retrosplenial and the temporo-parietal cortices, among others (Vogeley et al., 2001; Saxe and Kanwisher, 2003; Harris et al., 2005; Mitchell et al., 2005; Schiller et al., 2009), by using mostly, though not exclusively, verbal stimulus material (Walter et al., 2004; Freeman et al., 2010a).

While these studies obviously focus on types of knowledge and reasoning we all use extensively in our everyday life, it has become increasingly clear that other ways of processing person-related information are equally or sometimes even more significant. For example, individuals with high functioning autism, who can pass explicit experimental false belief tasks as well as controls, are still impaired in their daily social life and are unable to transfer this knowledge into more complex and ecologically valid situations (Klin et al., 2003). The remaining impairments are supposed to relate to difficulties in spontaneously attending to socially meaningful stimuli in real world environments and in experiencing social stimuli as significant (Klin et al., 2003; Senju et al., 2009; Kuzmanovic et al., 2011). This example highlights the importance of taking into account influences of the affective value of social cues in a comprehensive investigation of person perception. While the outcome of the initial categorization and of inferential analyses of person-related information also crucially affects subsequent evaluations and behavior toward the target person (Freeman and Ambady, 2011), this category and inference-dependent influence can be distinguished from affective influences of salient social cues on the decoder. Such affective and motivational effects are present before categorical social knowledge has fully developed (Farroni et al., 2002), and can act independently of top-down attention control (see next section).

## Affective influences of person-related information on the decoder

In general, humans' decision making is influenced by emotional factors (Slovic and Peters, 2006; De Martino et al., 2008). Such influences can be triggered by stimuli that have a predispositional or primary affective value, such as food and social cues including attractive faces or emotional expressions (Aharon et al., 2001; Bray and O'Doherty, 2007; Lin et al., 2012). Alternatively, stimuli can acquire an affective value through classical conditioning (also “Pavlovian conditioning”), i.e., when neutral stimuli acquire a positive or negative value due to repeated pairing with other unrelated positive or negative stimuli (Hermans et al., 2002). Especially during person perception, social cues are necessarily present and can influence the decoder due to their intrinsic affective value. This influence may concern an enhanced and prioritized processing relating to selective attention to, and recognition and representation of stimuli. Furthermore, social cues may also function as incentives and thus lead to reward-dependent learning.

Regarding the influence related to prioritized processing, neuroimaging studies have shown that face processing is enhanced for emotionally expressive as compared to neutral stimuli as a result of the modulatory influence of the amygdala (Vuilleumier and Pourtois, 2007). Known to play a central role in detecting affectively significant sensory input (Sander et al., 2003), the amygdala is able to modulate activity in brain networks associated with visual face processing and with other cognitive and affective responses in favor of the more salient emotional information via massive reciprocal connections (Vuilleumier and Pourtois, 2007; Pessoa, 2008; Robinson et al., 2010). Consistent across a line of studies, non-verbal person-related information including facial and bodily expressions as well as invariant facial features such as attractiveness or race elicits enhanced activity in the amygdala (Phelps et al., 2000; Hariri et al., 2002; Lieberman et al., 2005; Sergerie et al., 2008; Kuzmanovic et al., 2012). Moreover, increased activation of the amygdala has also been found for social stimuli in general, i.e., for neutral stimuli too and is thus independent of their valence, when compared to non-social stimuli (Vrticka et al., 2012). Critically, amygdala-driven modulation of visual processing by emotional information is also detectable for non-attended stimuli, i.e., without voluntary control or conscious awareness (Vuilleumier and Schwartz, 2001; Vuilleumier et al., 2001). Moreover, patients with lesions in the visual cortex who cannot achieve conscious visual experience can still discriminate facial expressions of emotions—presumably via a subcortical circuit including the superior colliculus, thalamus, and the amygdala (Adolphs, 2002). Similarly, autonomic measures demonstrate that patients with prosopagnosia, who are unable to recognize familiar faces, can still discriminate familiar from unfamiliar faces on an unconscious level (Ellis and Lewis, 2001). Thus, this subcortical processing may mediate affective influences independently of the activation of category-organized knowledge structures.

The described sensitivity of the amygdala to salient stimuli may play a central role in attracting the attention toward meaningful social cues. When this function is impaired, as in patients with amygdala lesions, spontaneous recognition of emotional expressions of faces is reduced (Adolphs et al., 2002), unless these patients have been explicitly instructed to attend to the informative eye region (Adolphs et al., 2005). Furthermore, in contrast to healthy or hippocampus-lesioned persons, patients with amygdala lesions do not demonstrate increased activation in visual face-related brain regions for fearful relative to neutral faces, but they show increased activation in these regions when faces are presented in a task-relevant relative to task-irrelevant position (Vuilleumier et al., 2004). Hence, the top-down attentional modulations by task demands can act independently of the modulations by affective significance of the stimuli via the amygdala (Vuilleumier and Pourtois, 2007).

Beyond these general affective influences related to prioritized processing, social cues can modulate affective responses to unrelated, but simultaneously or subsequently presented stimuli. Extending prior neuroimaging findings that face attractiveness or gaze following induce activity in reward-associated neural areas such as the orbitofrontal cortex and the ventral striatum (Aharon et al., 2001; Kranz and Ishai, 2006; Schilbach et al., 2010), pleasant social stimuli were also able to establish affective values in abstract and initially neutral stimuli by means of classical conditioning (Bray and O'Doherty, 2007). Thus, just like other types of reward such as food or money, person-related information can influence our evaluations of arbitrary stimuli when paired with them. Although the study by Bray and O'Doherty refers to classical conditioning and demonstrates the involvement of the ventral striatum, which has previously been associated with learning based on this principle, the fact that the measured effect relates to an evaluative attitude and not only to an affective response calls for a more precise reference to the similar, but not identical evaluative conditioning. This field of research provides further behavioral empirical evidence for influences of valent social stimuli such as likeable and dislikeable faces on the evaluation of neutral facial stimuli (Baeyens et al., 1992; Walther et al., 2005). In addition to their effect in classical and evaluative conditioning, which change the attitude and the affective reaction to previously neutral stimuli, positive and negative facial expressions were also shown to modulate complex consumption behavior. Subliminally presented smiling faces increased the consumption of and the willingness to pay for beverages while frowns had the opposite effect (Winkielman et al., 2005). Interestingly, these effects on overt behavior occurred without eliciting changes in conscious feelings. Such effects, “in which the motivational characteristics of a predictor influence the vigor of an action with respect to which it is formally completely independent are called “Pavlovian-Instrumental Transfer” (PIT) (Talmi et al., 2008, p. 360). The PIT has been shown to be mediated by the ventral striatum and the amygdala, in concordance with the regulatory role of these regions in integration of affective-motivational, cognitive, and motor processing in the brain (Talmi et al., 2008).

Taken together, these findings suggest that humans are equipped with motivational predispositions to respond to person-related information, among other biologically relevant stimuli, presumably due to its significance for adaptive social behavior and, in consequence, survival (Dunbar, 2009). In consequence, social cues may be more efficiently detected for the purpose of prioritized processing, memorization, and evaluation (Klin et al., 2003; Vuilleumier and Pourtois, 2007). Furthermore, this affective or motivational value of social cues can also influence simple approach-avoidance (Chen and Bargh, 1999) and complex instrumental behaviors (Winkielman et al., 2005) via reward-dependent learning.

## Processing differences for distinct formats of person-related information

While the basic sensory and cognitive processing of verbal and non-verbal information is generally associated with distinct neural areas, there are also format-dependent differences specifically related to social processing. From very early on, psychological theories of interpersonal communication assumed that non-verbal information has been assumed to have a greater impact on the affective, relational level of communication (Watzlawick et al., 1967). Furthermore, linguistically encoded information always requires the processing of an explicit semantic code with a complex logical syntax and is thus necessarily linked to high-level cognitive processing, while non-verbal information lacks such an explicit interpretation code (Bente and Kraemer, 2008; Kraemer, 2008).

Recently, neuroimaging studies could provide empirical support for these assumptions by showing that the processing of non-verbal person information consistently recruits the amygdala (Hariri et al., 2002; Winston et al., 2002; Sergerie et al., 2008; Todorov and Engell, 2008; Todorov et al., 2008). By contrast, social inferences based on verbal descriptions of other persons' actions or traits, as well as explicit categorization of facial stimuli involved medial prefrontal, retrosplenial and temporo-parietal cortical brain regions (Mitchell et al., 2002, 2005; Harris et al., 2005; Schiller et al., 2009; Freeman et al., 2010a; Zaki et al., 2010). Only a few studies directly compared the processing of verbal and non-verbal information and further substantiated the view that different neural networks show relatively stronger links to the one or the other information format (Freeman et al., 2010b; Zaki et al., 2010; Kuzmanovic et al., 2012). For instance, during a person evaluation task, evaluations of increasing intensity based on non-verbal information recruited the amygdala to a greater extent, whereas the same pattern was observed in the retrosplenial cortex for verbal information (Kuzmanovic et al., 2012). This finding confirms qualitatively different cognitive processes underlying person perception with a closer relation of non-verbal information and salience-dependent processing on the one hand, and of verbal information and the high-level social cognition on the other.

In addition to the differences outlined for the information format, the context and the content of social cues modulate social processing in a complex manner. For instance, it has been supposed that social cognitive processes fundamentally differ when people engage in direct interpersonal interactions than when merely observing social cues, a topic that is beyond the scope of the present article [for a discussion on the second-person approach see Schilbach et al. (2013)]. Taken together, the exact format, context and content of the information that is available during person perception can critically determine the kind of cognitive processes recruited. While both inferential-categorical processing and affective influences may take place for all sources of information, possible effects have to be differentially weighted for distinct kinds of person stimuli.

## Integration of distinct but interactive person perception aspects

Although different processes with distinct functional implications can be delineated for person perception, they also have to be considered as embedded in a strongly interconnected neural network, thereby reciprocally modulating each other. For instance, the fact that person-related information is able to influence the observer due to its affective value does not mean that this influence is absolutely automatic and unfiltered, without the modulation by reflective and goal-directed processes such as voluntary attention and conscious intentions, appraisals and attitudes (Pessoa, 2008). Exactly this dynamic interactive nature of social processing including the influence of sensory cues has been described previously (Freeman and Ambady, 2011). However, in this model the impact of salient affective social cues is defined in terms of categories and corresponding stereotypes without taking into account their ability to prioritize processing and act as a reward, thereby constituting a category-independent source of influence on the beholder.

An example of the top-down modulation of affective responses to monetary rewards has been provided both on the behavioral and neural level. When people believe that their trading partner has a praiseworthy moral character, they rely less on the actual behavior resulting in monetary losses or wins (Delgado et al., 2005). On a neural level, this effect corresponds to reduced differential activity in the ventral striatum in response to positive and negative outcomes for trading partners with positive or negative moral character evaluations as compared to neutral partners (Delgado et al., 2005). Thus, personality trait inferences can greatly influence the reward-dependent, motivational responses within person perception. On the other hand, however, facial attractiveness and expression may influence deliberate high-level judgments about unrelated performances such as political votes or management success (Ballew and Todorov, 2007; Antonakis and Dalgas, 2009), an effect mediated by the amygdala (Rule et al., 2010).

Thus, it can be assumed that there are different concurrent and mutual modulations during person perception, arising from both top-down cortical attentional and inferential networks, as well as from bottom-up primary and secondary sensory areas (Freeman and Ambady, 2011). We argue, however, that the intrinsic affective and motivational value of social stimuli exerts an additional influence over the general person perception via subcortical regions associated with salience and reward processing.

## Conclusions

In the light of the diversity of findings relating to person perception, person-related information has to be regarded as a basis for inferences and categorizations as well as a source of a potential affective-motivational influence on the decoder. As a complex spontaneous constructive process, and not a simple one-to-one representation of available cues, person perception includes a huge amount of uncertainty and is thus prone to biases. In order to prevent oversimplifications and deficient interpretations of empirical findings, an integrative theoretical reflection on person perception should carefully consider affective and motivational effects of person-related information in addition to the dynamic of interactive cognitive processes previously described in integrative frameworks.

### Conflict of interest statement

The authors declare that the research was conducted in the absence of any commercial or financial relationships that could be construed as a potential conflict of interest.
